# The Mexican bean beetle (*Epilachna varivestis*) regurgitome and insights into beetle-borne virus specificity

**DOI:** 10.1371/journal.pone.0192003

**Published:** 2018-01-29

**Authors:** Cassidy R. Gedling, Charlotte M. Smith, Christophe M. R. LeMoine, Bryan J. Cassone

**Affiliations:** 1 Department of Plant Pathology, The Ohio State University, Wooster, OH, United States of America; 2 Department of Biology, Brandon University, Brandon, MB, Canada; Portland State University, UNITED STATES

## Abstract

For nearly 400 million years, insects and plants have been embattled in an evolutionary arms race. Insects have developed diverse feeding strategies and behaviors in an effort to circumvent and overcome an extensive collection of plant defense tactics. Sap-sucking insects often inject saliva into hosts plants, which contains a suite of effector proteins and even microbial communities that can alter the plant’s defenses. Lacking salivary glands, leaf-feeding beetles represent an interesting group of phytophagous insects. Feeding beetles regurgitate onto leaf surfaces and it is thought that these oral secretions influence insect-plant interactions and even play a role in virus-vector specificity. Since the molecular and biological makeup of the regurgitant is virtually unknown, we carried out RNA sequencing and 16S rDNA analysis on a major soybean pest, *Epilachna varivestis*, to generate the first ever beetle “regurgitome” and characterize its microbiome. Interestingly, the regurgitant is comprised of a rich molecular assortment of genes encoding putative extracellular proteins involved in digestion, molting, immune defense, and detoxification. By carrying out plant inoculation assays, we reinforced the fundamental role of the regurgitant in beetle-borne virus specificity. Ultimately, these studies begin to characterize the importance of regurgitant in virus transmission and beetle-plant interactions.

## Introduction

The interactions between host plants and their phytophagous insects are intrinsically complex and subject to remarkable evolution, where both have adapted strategies to avoid each other’s defense systems. Plants have developed an extraordinary array of physical barriers, constitutive chemical mechanisms, and direct and indirect inducible defenses intended to counter/offset the effects of insect attack [1–5]. In parallel, insects have adapted tactics to combat the diverse arsenal of plant defenses, allowing them to feed, grow, and reproduce on their host plants [6]. A wide range of phytophagous insects possess highly modified piercing-sucking mouthparts, enabling them to use phloem sap as their exclusive food source. During the feeding process, saliva is injected into plant tissues to aid in penetration, ingestion of nutrients, and modulate plant responses [7]. Moreover, a subset of secreted proteins with structural, chelating, or enzymatic properties is thought to serve as the ‘effectors’ of these processes [8,9].

Leaf-feeding beetles (Order Coleoptera) possess “chewing” mouthparts consisting of two opposing mandibles, which are used to remove leaf sections or entire leaves. Lacking the salivary glands of their sap-sucking counterparts, these beetles are thought to regurgitate onto the surface of leaves while feeding to begin the digestion process. The bulk of the regurgitant likely originates from the foregut, though the gnathal glands in the cephalic regions may also contribute [10,11]. Importantly, the deposited regurgitant is believed to be a major source of effector proteins and/or small molecules [8] that can evoke changes in host plant defenses, thereby making the plant more vulnerable to the herbivore attack [9]. Such effectors have been well documented in the saliva of sap-sucking insects [12–21] but little information is currently available for the regurgitant of chewing insects. In addition to effectors, recent studies have indicated these oral secretions contain diverse microbial communities that may alter plant-insect interactions [22,23]. Taken collectively, the regurgitant of leaf-feeding beetles appears intricate and multifaceted but is only beginning to be explored.

Beetles vector at least six groups of plant viruses: *Machlomovirus*, *Bromovirus*, *Carmovirus*, *Comovirus*, *Sobemovirus*, and *Tymovirus*. A unique and specific relationship exists between herbivore beetles and the plant viruses they vector: viruses that are transmissible by beetles are solely transmitted by beetles. Inoculative beetles deposit the active virus in regurgitant on the surface of the wounded leaf during feeding [24]. Unlike most other plant viruses, beetle-borne viruses can be inoculated into a chewing wound. This is because the virus particles are rapidly translocated in the xylem elements away from the inoculation site and infect unwounded cells at a distance from the feeding site [25,26]. Previous work has revealed that factor(s) in the regurgitant also play a role in the virus-vector specificity [11,27,28]. Indeed, mixing purified virus with regurgitant prevents host plant infection by non-beetle-borne viruses but has no effect on beetle-transmissible viruses. Though this specificity is well established, little is known regarding the specific factor(s) in the regurgitant that govern the selective inhibition.

One of the most prevalent and destructive leaf-feeding beetles in North America is the Mexican bean beetle, *Epilachna varivestis* Mulsant [29]. Native to the plateau region of southern Mexico, *E*. *varivestis*’ can now be found from Guatemala to southern Canada [30,31]. Since its establishment in the United States in 1942, the beetle has become a major economic pest of *Phaseolus* spp., including soybean (Glycine max (L.) Merr.) [31,32]. *E*. *varivestis* is also an efficient vector of several plant viruses, including *Cowpea severe mosaic comovirus* [29], *Southern bean mosaic sobemovirus* [33], and *Black gram mottle carmovirus* [34]. Perhaps the most important virus vectored by the beetle is *Bean pod mottle virus* (BPMV). This is a positive-sense single-stranded RNA comovirus, and one of the most ubiquitous viruses of soybean in North America.

To date, little is known about the molecular, chemical, or biological composition of beetle regurgitant or how the factor(s) within may modulate plant-insect interactions. To begin to characterize this, we carried out RNA sequencing on the oral secretions of *E*. *varivestis* to assemble the first ever leaf-feeding beetle “regurgitome”. We also implemented 16 rDNA sequencing to characterize the bacterial communities in the regurgitant. The regurgitant consisted of a rich molecular assortment of genes encoding putative extracellular proteins involved in digestion, molting, immune defense, and detoxification. By carrying out plant inoculation assays that combined purified virus and regurgitant, we reinforced the importance of regurgitant in the unique and specific relationships between leaf-feeding coleopterans and the viruses they transmit. Ultimately, these studies begin to characterize the critical role of regurgitant in virus transmission and in the interactions between leaf-feeding beetles and their host plants.

## Materials and methods

### Beetle colony maintenance

A laboratory colony of *Epilachna varivestis* was established in 2014 from multiple, ongoing field collections in Ohio and reared in growth chambers under controlled conditions of 25 ± 3°C, 65% RH with a 14-h:10 h light-dark cycle that included 1.5 h dawn and dusk transitions. Beetles were maintained on ‘Sloan’ seedlings placed in 47.5 cm x 47.5 cm x 47.5 cm cages.

### The *E*. *varivestis* regurgitome

#### Regurgitant collection

Regurgitant was isolated from adult feeding beetles (between 1–3 weeks old) at leaf wounding sites using capillary glass tubes, and immediately placed into 0.5 mL microcentrifuge tubes containing a 5:1 ratio of extraction buffer XB (Thermo Fisher Scientific, Waltman, MA) and 2-Mercaptoethanol (Sigma-Aldrich, St. Louis, MO). Samples were then snap frozen in liquid nitrogen and stored at −80°C until nucleic acid isolation.

#### Nucleic acid isolation

Total RNA and genomic DNA (gDNA) was extracted from approximately 200 *E*. *varivestis* by implementing a multifaceted approach. First, gDNA was isolated by following the DNA purification protocol of the AllPrep DNA/RNA Mini Kit (QIAGEN, Germantown, MD). The initial flow-through from the DNA spin column (step 5) was then collected for RNA extraction using the Arcturus PicoPure RNA Isolation Kit (Thermo Fisher Scientific, Waltman, MA), following the manufacturer’s protocol but with the omission of LCM Caps. RNA and gDNA quality was evaluated using the Nanophotometer NP80 (Implen Inc., Westlake Village, CA), and quantity was calculated on the Qubit 3.0 fluorometer using the RNA HS or dsDNA HS assay kits (Thermo Fisher Scientific, Waltman, MA).

#### cDNA library preparation

RNA (500 ng/sample) was used to generate one cDNA library for RNA sequencing using the TruSeq Sample Prep Kit V1 (Illumina, San Diego, CA) following the company recommended protocols. Quantity and quality of the cDNA library was assessed using the BioAnalyzer 2100 (Agilent Technologies, Santa Clara, CA) and then diluted to 100 fmoles.

#### Illumina sequencing

The cDNA and 16S rRNA libraries were sequenced in 300-bp paired-end fashion on one run of the llumina MiSeq System at the Génome Québec Innovation Centre. Illumina Analysis Package CASAVA 1.8.2 was used to perform bcl conversion and demultiplexing. Image deconvolution and quality value calculations were carried out using the Illumina GA pipeline v1.6.

#### Regurgitome assembly

Raw reads of the cDNA library were imported into CLC Genomics Workbench (v6.5.1, CLC Bio) and trimmed for quality, adapter indexes and poly(A) tails using the default settings (Ambiguous limit = 2, quality limit = 0.05). Processed reads were assembled *de novo* into contigs using two independent approaches. First, using the CLC Bio algorithm based on de Bruijn graphs and the optimized parameters: Word Size = 64, Bubble Size = 900, Length Fraction = 0.65 and Similarity Fraction = 0.85. Second, using Oases v0.2.08 [35] with Kmer sizes of 53, 59, 65, 71, 77, 83, and 89. To obtain the set of non-redundant transcripts for each assembly, transcripts ≥80% sequence similarity were collapsed into clusters and the longest read retrieved using CD-HIT-EST [36]. The assemblies were then merged into a final assembly using Minimus2 [37]. Only contigs of ≥300 nt in length with average coverage ≥5 were included in the final assembly.

Microbial contamination was identified and removed from the *E*. *varivestis* transcriptome using desktop-downloaded BLASTn against the NCBI bacteria non-redundant database (*E*-value <1 x 10^−50^) and a GC content threshold of 45%. Contigs of soybean origin were identified and removed using BLASTn against the most recent *Glycine max* [Glyma2.0] reference genome including scaffold sequences (*E*-value <1 x 10^−50^). The remaining contigs were assigned hierarchical gene ontologies (GO terms) on the basis of biological processes, molecular functions and cellular components using the platform-independent Java™ 6 implementation of the BLAST2GO software [38]. The top five BLASTx hits to the nr database with a cut-off *E*-value of 10^−3^ were considered for GO annotation.

#### Pair-wise comparisons among arthropods

Pair-wise comparisons of *E*. *varivestis* regurgitome contigs to the gut transcriptome assemblies of seven arthropod species across five orders were carried out using desktop downloaded tBLASTx software with a set *E*-value threshold of 10^−10^. The complete list of species included *Anopheles gambiae* (malaria mosquito, order Diptera, 22,889 sequences) [39], *Pectinophora gossypiella* (pink bollworm, order Lepidoptera, 11,746 sequences) [40], *Haemaphysalis flava* (hard tick, order Ixodida, 76,556 sequences) [41], *Periplaneta americana* (American cockroach, order Blattodea, 78,837 sequences) [42], *Leptinotarsa decemlineata* (Colorado potato beetle, order Coleoptera, 21,622 sequences) [43], *Chrysomela tremulae* (poplar leaf beetle, order Coleoptera, 10,876 sequences) [44], *Gastrophysa viridula* (green dock leaf beetle, order Coleoptera, 20,791 sequences) [45].

#### Identification of putative secreted proteins

The six open reading frame (ORF) amino acid sequences were predicted from the *E*. *varivestis* contig sequences using ORF-Predictor [46]. Only the subset of predicted sequences ≥50 amino acids was used in subsequent analyses. The SignalP 4.1 neural networks algorithm [47] was implemented to detect putative transmembrane proteins with signal peptide secretion and cleavage site signatures in their amino acid sequences using the default settings for D-score.

#### 16S rDNA library preparation and analysis

Bacterial 16S rRNA genes present within the beetle regurgitant were amplified using universal bacterial primers with the appending of Illumina adapter sequences to construct an amplicon library from the V3-V4 region of the 16S rDNA genes. By using the Illumina polymerase-binding regions, samples can be sequenced in lieu of sequencing primers thereby eliminating the need for an additional ligation step. The primer pairs, retrieved from [48], were: (A): S-D-Bact-0341-b-S-17, 5’-CCTACGGGNGGCWGCAG-3’ and S-D-Bact-0785-a-A-21, 5’-GACTACHVGGGTATCTAATCC-3’ [49]; and (B): S-D-Bact-0008-a-S-16, 5’-AGAGTTTGATCMTGGC-30 [50] and S-D-Bact-0907-a-A-20, 5’-CCGTCAATTCMTTTGAGTTT-3’ [51]. PCR was performed at an initial denaturation temperature of 96°C for 3 min, followed by 25 cycles of 96°C for 30 s, 55°C for 30s and 72°C for 30 s. A final elongation step at 72°C was run for 5 min. PCR products were purified using 20 μL of AMPure beads (Beckman Coulter, Takeley, UK) and quantified on the Qubit 3.0 fluorometer.

Taxonomy was assigned to amplicon sequences *in* the 16S rDNA library using a high performance version of the RDP Naïve Bayes taxonomic classification algorithm via the BaseSpace 16S Metagenomics pipeline.

### BPMV-*E*. *varivestis* specificity assays

The experiments described below were largely developed based on the previously established protocols (see [11,28]). One beetle transmissible virus, *Bean pod mottle virus* (BPMV) and one beetle non-transmissible virus, *Soybean mosaic virus* (SMV) were used in these experiments.

#### Virus extraction and isolation

Soybean leaves infected with BPMV or SMV were homogenized in 10mM KHPO_4_ buffer (pH 7). The homogenate was clarified overnight by low speed centrifugation with 8% butanol at 4°C. After a centrifuge at 13,000 × *g* for 20 min, virus particles were precipitated from the supernatant by using an equal volume of 16% PEG (Sigma Chemical Co., St. Louis, Mo.) with 0.5 M NaCl. After precipitation, the pellet was resuspended in 5 ml of KHPO_4_ buffer and incubated at room temp for 10 min. The resuspended pellet was transferred to a 15 ml polypropylene centrifuge tube containing 1.2 ml of chloroform, and centrifuged at 12,000 × *g* for 5 min. The top aqueous layer (containing the RNA) was precipitated by addition of 0.5 volume of isopropanol for 5 min at room temp, followed by centrifuging at 5,000 × *g* for 5 min. The resultant pellet washed with 80% ethanol and resuspended in washing buffer (10 mM Tris-HCl [pH 7.5], 0.15 M LiCl, 1 mM EDTA). This process was repeated 3 times with the final resuspension in 1 mL of KHPO_4_ buffer.

#### Regurgitant collection

Beetles were induced to regurgitate by holding the individual between the thumb and forefinger and gently prodding the mouthparts with a capillary glass tube, which collected the regurgitant. Only freshly collected regurgitant were used for the experiments.

#### Inocula

The ‘Sloan’ cultivator (susceptible to BPMV and SMV) was used to assess the impact of *E*. *varivestis* regurgitant on virus infectivity using two different inoculation approaches: mechanical leaf-rub inoculations and gross wounding (described below). Inoculations were carried out for each virus independently using a predetermined dose of virus that gave >90% infection of the positive control for the mechanical and gross wounding assays, respectively. The inocula deposited at the leaf wounding sites consisted of the purified virus in 0.01 M KHPO_4_ mixed with five-fold dilutions of regurgitant (4 in total). After 14 d of visual assessment of symptom development, virus infection was confirmed and titer estimated in using enzyme-linked immunosorbent assays [52]. A total of 30 replicates were carried out for each combination of virus/regurgitant mixture.

#### Mechanical leaf-rub inoculations

Following the protocols outlined in [53], isolated virus was mixed with and mechanically inoculated onto the youngest trifoliate of 1.5 wk old test plants lightly dusted with corundum. Rub-inoculation of plants with isolated virus and KHPO_4_ buffer served as a positive control.

#### Gross wounding assays

A single hole 8 mm in diameter was bored into a leaf of the test plant using the fractured edge of a glass cylinder. Immediately prior to cutting the hole, the cylinder was dipped into an equal volume mixture of isolated virus and regurgitant. Control plants underwent identical treatment, but with the inoculum consisting of an equal volume mixture of isolated virus and KHPO_4_ buffer.

#### Statistical analysis

To test the statistical significance of differences in the relative proportions of infected plants for each virus and ratios of regurgitant to purified virus mixture, we subjected the data to a chi-square analysis followed by a 2 x 24 Marascuilo procedure [54], with a threshold of significance at *P* < 0.05.

## Results and discussion

### Assembly of the *E*. *varivestis* regurgitome

A cDNA library derived from the regurgitant of roughly 200 *E*. *varivestis* individuals was sequenced, which produced 56,289,018 paired end reads of 300 nt. After trimming (quality, adapters, and poly (A) sequences) 40,118,669 reads were obtained. The unprocessed reads have been deposited in the NCBI Sequence Read Archive (SRA) under the accession SRR4934939 (study SRP092603). *De novo* assembly of the trimmed reads generated 104,977 non-redundant contigs of >300 nt with a mean length of 625 nt. Subsequent removal of bacterial (n = 38,352) and soybean (n = 21,077) contamination resulted in 45,548 contigs considered to be beetle in origin, and this subset represents the *E*. *varivestis “*regurgitome”. These contigs are thought to be derived mostly from the beetle’s gastrointestinal cell products. While the regurgitome is presumably similar in composition to the gut transcriptome, our approach has one important distinction: the regurgitome is likely produced by enterocytes lining the lumen side of the gastrointestinal tract, which play important roles in secretion and likely expresses the transcripts encoding for host plant modulating effector proteins. In contrast, assembly of the gut transcriptome would have yielded a mixed transcriptomic profile, the bulk of which is comprised of structural, muscular, and absorptive cells of little significance for the regurgitant.

### Comparisons to gut transcriptomes of diverse arthropods

**Fig 1:**
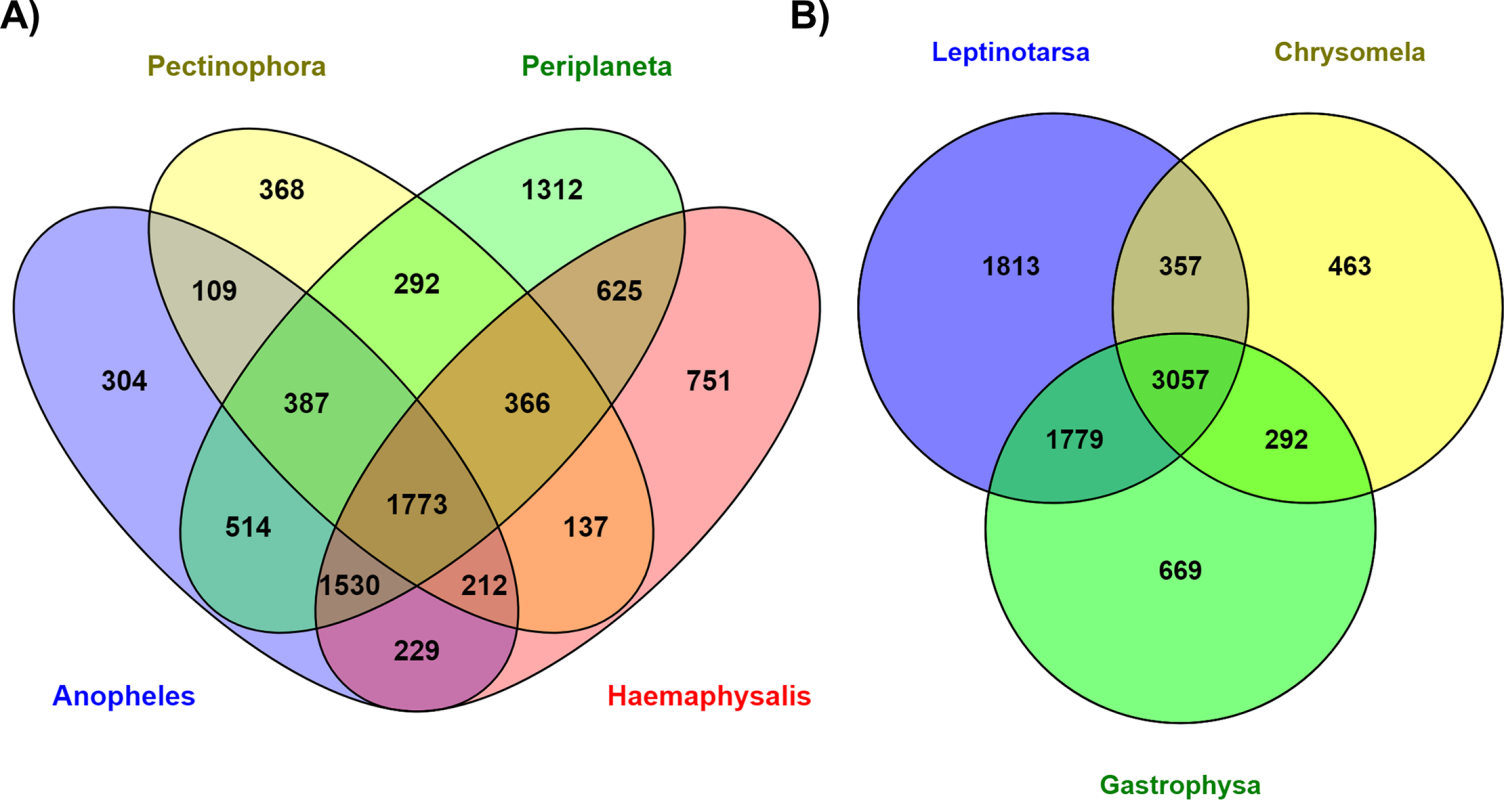
Venn diagram [84] showing tBLASTx (E-value <10^−10^) pair-wise ortholog matches of the *E*. *varivestis* to the gut transcriptomes to the characterized gut transcriptomes. (A) four arthropod species with diverse feeding strategies (*Pectinophora gossypiella*, *Haemaphysalis flava*, *Periplaneta Americana*, *Periplaneta americana*); and (B) three herbivore Coleopteran beetles (*Leptinotarsa decemlineata*, *Chrysomela tremulae*, *Gastrophysa viridula*).

We surmised that the *E*. *varivestis* regurgitome would show extensive similarity to the gut transcriptomes of other arthropod species (see above), particularly phytophagous Coleopterans. To investigate this, we first wanted to compare the Mexican bean beetle regurgitome to the gut transcriptomes of arthropods displaying diverse feeding strategies. This included blood-feeding mosquitoes and ticks (*Anopheles gambiae* and *Haemaphysalis flava*), a herbivore caterpillar (*Pectinophora gossypiella*), and a generalist cockroach (*Periplaneta americana*). The pair-wise comparisons revealed that less than 20% (n = 8,909) of *E*. *varivestis* contigs had a significant match to at least one of the four arthropod transcriptomes (*E*-value <10^−10^), with only 1,773 contigs common to all species (Fig 1A). Next, we sought to determine if the *E*. *varivestis* regurgitome carried considerably more commonality to the gut transcriptomes of herbivore Coleopteran beetles. This included two leaf-feeding beetles (*Leptinotarsa decemlineata* and *Chrysomela tremulae*) and a grass-feeding (*Gastrophysa viridula*) species. Similar to the previous analysis, <20% of *E*. *varivestis* contigs (n = 8,430) matched one or more of the beetle transcriptomes, with 3,057 contigs shared by all species (Fig 1B).

Our pairwise comparisons indicated that only a small proportion of the *E*. *varivestis* regurgitome had significant sequence homology to the gut transcriptomes of a diverse grouping of arthropod species. Most of the 8,430 *E*. *varivestis* contigs matching other Coleopteran beetle(s) also matched at least one of the other arthropod species (~80%). This result was quite surprising given the greater evolutionary relatedness and similarity in feeding strategies among the beetle species. While it is probable that the *E*. *varivestis* regurgitome contains many genes encoding proteins that carry out unique digestive and defensive functions for *E*. *varivestis* and perhaps other Coccinellidae species, this result is likely partially attributed to sequencing coverage of our contrasting species. The Coleopteran gut transcriptomes were constructed using 454-mediated pyrosequencing and 0.28 to 1.24 million reads per species, whereas the gut transcriptomes of the other arthropods were generated using Illumina sequencing and between 18.6 to 223 million reads per species. Thus, the evolutionary relatedness among Coleopterans was probably offset by the deeper sequencing coverage of the other species. Constructed from nearly 57 million paired-end Illumina reads, the *E*. *varivestis* regurgitome thus represents the deepest coverage and most targeted effort to catalogue potential beetle effectors.

### Identification of genes encoding putative extracellular proteins

Beetle effectors are proteins secreted into the cells of host plants during feeding. Therefore we initiated our search for putative *E*. *varivestis* effectors by translating the 45,548 contigs into their putative amino acid sequences. ORF analysis predicted 34,835 (76.5%) encoded peptide sequences of ≥ 50 AA. We then carried out *in silico* analysis on the peptide sequences using the SignalP server. A total of 1,555 sequences were predicted to have a secretion signal (S1 Table). This Transcriptome Shotgun Assembly project has been deposited at DDBJ/EMBL/GenBank under the accession GFPI00000000. The version described in this paper is the first version, GFPI01000000.

**Fig 2:**
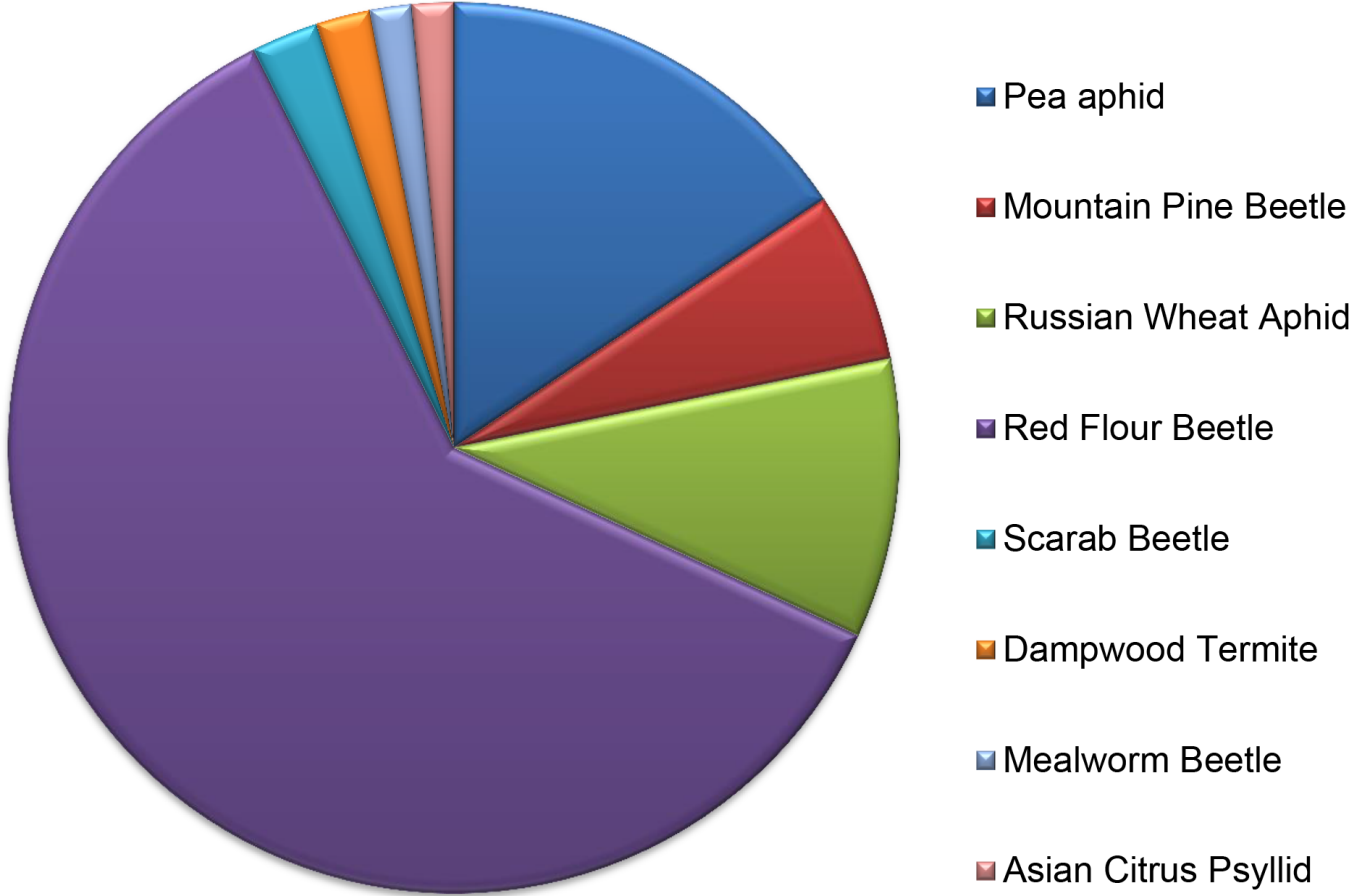
BLASTx top ortholog matches to organisms within the NCBI non-redundant (nr) database for the subset of 992 *Epilachna varivestis* contigs encoding putative secreted proteins with a significant BLASTx match (E-value <10^−3^). Only organisms with ≥10 matches are shown.

For the subset of 1,555 contigs encoding putative secreted proteins, 992 (64%) had a significant BLASTx matches to the nr database. Fig 2 shows the top ortholog matches to organisms within the nr database. Not surprisingly, the largest number of significant matches were to a Coleopteran model organism, *Tribolium castaneum*, with >40% (n = 401) of all top matches. Other top matches include two other Coleopterans, *Oryctes borbonicus* (n = 16) and *Dendroctonus ponderosae* (n = 41) as well as two aphid species, *Acyrthosiphon pisum* (n = 101) and *Diuraphis noxia* (n = 67). The relatively large number of matches to aphids may be indicative of some commonality in the composition of secreted proteins in the salivary glands of sap-sucking insects and the regurgitant of beetles.

### Functional characterization of putative effector proteins

**Fig 3:**
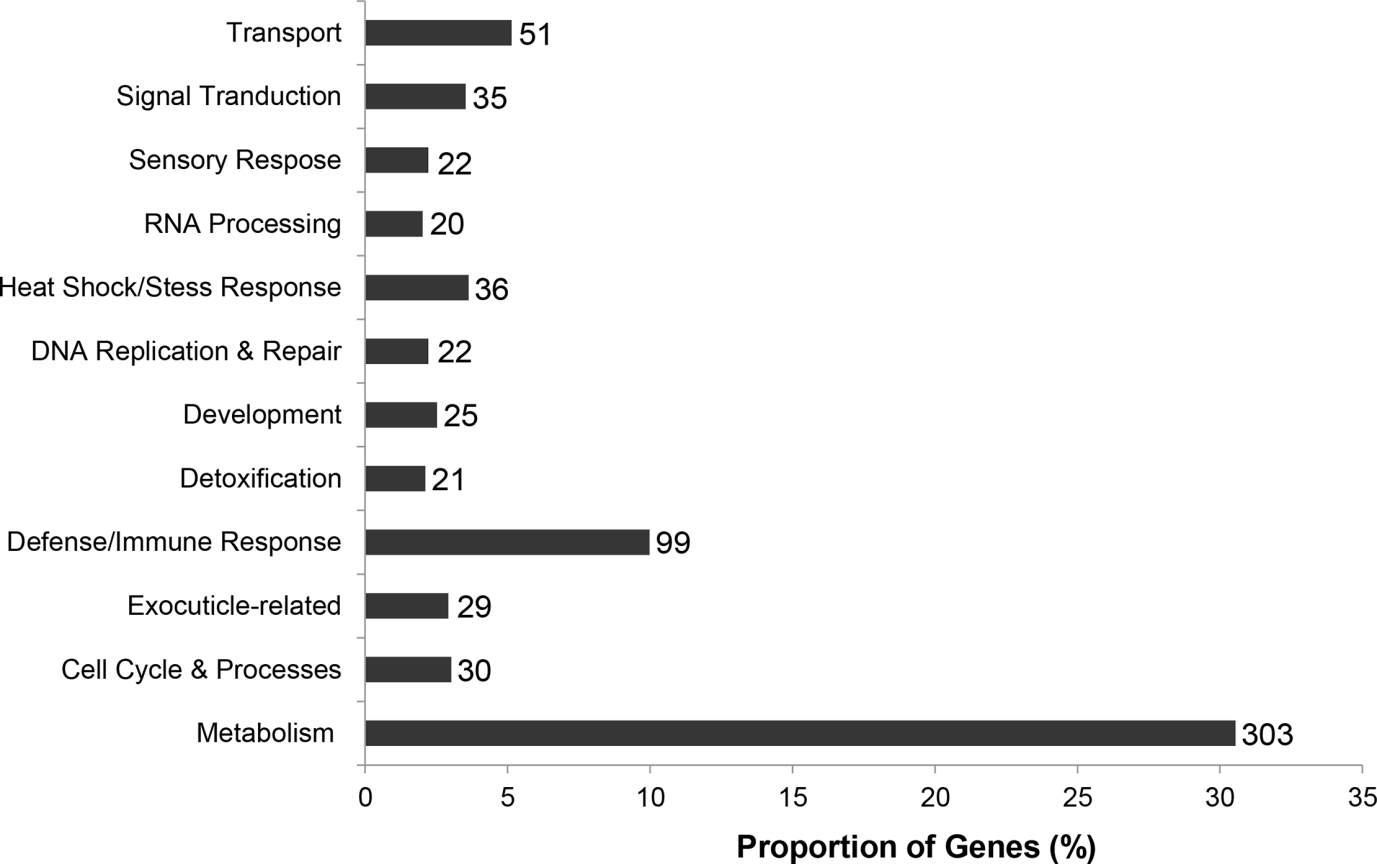
Distribution of *Epilachna varivestis* contigs encoding putative secreted proteins among functional categories. Bars indicate the proportion of genes in each category: Number of genes in each category is given beside each bar. Percentages do not total to 100 as not all categories are shown.

Based on the gene ontologies (biological processes, molecular functions, and cellular components) assigned by BLASTx, contigs were manually placed into various categories and sub-categories based on consensus function. This was not possible for 23% of genes (n = 229), as they could not be assigned any putative function. The proportion of differentially expressed genes in twelve functional categories containing of a minimum of 20 genes is given in Fig 3 (other categories not shown). A complete description of the annotation for each gene can be found in S2 Table. The most populous categories was “Metabolism” containing 40% of genes (n = 303) and “Defense/Immune Response” (n = 99).

Over the past few years, a large body of research has accumulated describing the molecular and chemical makeup of salivary glands and saliva of various sap-sucking insects [16,55–57]. On the other side of the spectrum, virtually no studies have been undertaken to describe the composition of regurgitant in leaf-feeding beetles. The only data available are enzymatic assays indicating that the beetle regurgitant is rich in proteases [58] and ribonucleases [59]. Our study largely supports this at the molecular level by identifying a considerable number of genes encoding these enzymes in our dataset. However, as described below, *E*. *varivestis* regurgitant appears to also contain an extensive suite of other proteins involved in gastrointestinal, immunological, and developmental processes.

### Regurgitant genes are involved in digestion

**Fig 4:**
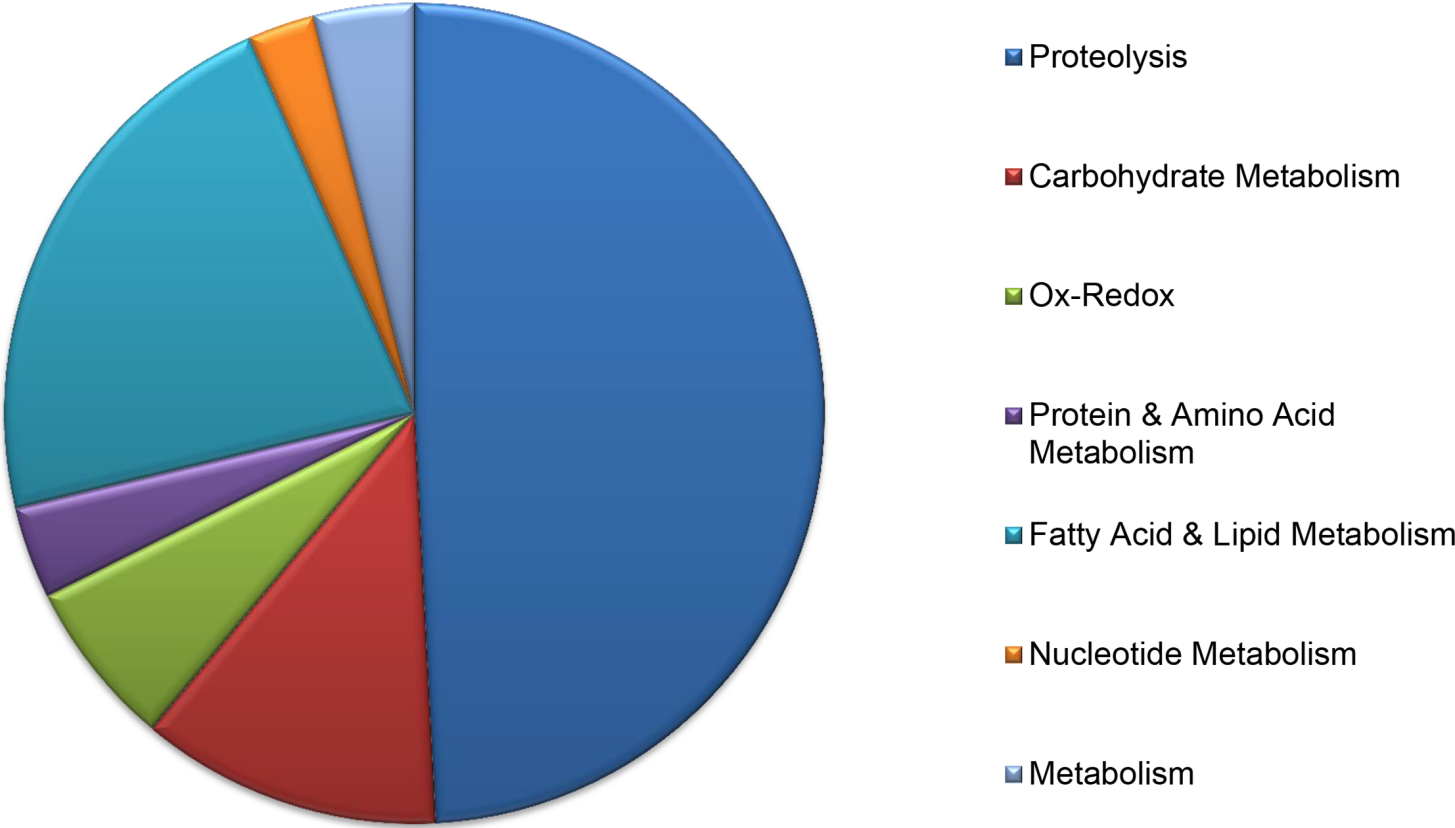
Distribution of *Epilachna varivestis* contigs encoding putative secreted proteins involved in metabolism.

Lacking the salivary glands of sap-sucking insects, it has been speculated that beetles regurgitate oral secretions onto the leaves to begin their digestive processes. Our findings of a large number of regurgitant genes encoding putative secreted proteins involved in digestion reinforces this idea. Nearly half of metabolic genes encode proteins with proteolytic activity (47%), of which the vast majority function as lysosomal proteases (e.g., cathespins) (Fig 4). Lysosomes are intracellular organelles that play key roles in the digestive breakdown and recycling of diverse biological materials [60]. For herbivorous beetles, this includes the enzymatic break down of proteins in leaves into smaller peptides and amino acids that can be readily absorbed and utilized by the organism. The next most populous sub-categories within Metabolism were fatty acid/lipid and carbohydrate related. The former included an array of lipases, which are one of the main digestive enzymes involved in the insect digestion process [61]. Carbohydrate metabolism consisted of a variety of enzymes involved in degradation complex carbohydrates, such as amylases, maltases, and glycosyl hydrolases, some of which may also play an important role in breaking down plant cell walls [62–64]. The transport of molecules across cell membranes and between subcellular compartments is also an essential component of both digestion and normal cellular functions. Interestingly, our dataset produced several gene products associated with the transport of simple molecules, such as sugars and vitamins, and with vesicular trafficking which are essential for normal digestive function [65,66].

### Regurgitant genes function in defense responses and detoxification

For nearly 400 million years phytophagous insects and their host plants have been entwined in an evolutionary arms race [9]. Plants have developed different mechanisms to fend off or deter insect attack, whereas insects employ a multitude of strategies to overcome these plant barriers [6]. Evidence has quickly accumulated that indicates sap-sucking insects secrete proteins and/or small molecules in their saliva to suppress host plant defenses [12–17,19–21]. Lacking salivary glands, it is likely that herbivore beetles secrete these proteins in their regurgitant. Supporting this, we found approximately 13% of the genes encoding putative extracellular proteins function in defense/immune. This includes an array of attacins, defensins, toll-pathway genes, C-type lectins, glutathione peroxidase, 1,3-beta-D glucan binding proteins, and autophagy genes [67]. Several other genes are involved in detoxification processes, such as cytochrome p450s and a variety of esterases. Many of these genes likely aid in the detoxification and even sequestration of plant chemical defenses [68]. Overall this subset serves as strong candidates for effectors that play active roles in combatting the anti-herbivory defenses of soybean. Functional assays targeting our most promising candidates will better implicate specific defense and detoxification genes in modulating plant-insect interactions.

### Regurgitant contains genes involved in exocuticle molting

One of the larger categories contained genes involved in exocuticle-related functions, namely cuticle proteins and chitinases. In insects, these proteins belong to family 18 glycosyl hydrolases, and have been detected in gut tissues [69]. They are predicted to mediate the digestion of chitin present in the exoskeleton chitooligosaccharides [70,71]. The *E*. *varivestis* life cycle consists of an egg stage followed by four instar stages and a pupal stage over a 30 to 70 d period before emerging as adults. Since it is unlikely that these the regurgitant plays a role in molting, these genes are probably highly expressed throughout the organism and are therefore also present in the regurgitome.

### Microbiome of the regurgitant

A large body of literature has amassed showing many insect groups are colonized by communities of diverse microbes, some of which act as symbionts [72–76]. There is growing evidence indicating these symbioses in the saliva of sap-sucking insects play an important role in host plant interactions [77–79]. Recent studies have demonstrated some chewing insects orally secrete symbiotic gut bacteria onto the surface of wounded leaves sites during feeding. Remarkably, these microbes can manipulate plant physiology to the benefit of their insect host in terms of nutrient acquisition [22,23]. This prompted us to carry out 16S rDNA amplicon sequencing in an effort to catalogue the bacterial communities in the regurgitant of *E*. *varivestis*. We identified a total of 1,230 bacterial species representing 577 genera (S3 Table), suggesting the regurgitant is comprised of a diverse microbiome.

The *E*. *varivestis* regurgitant contained a multitude of microbes that can be considered candidates for modulating plant physiology. By applying bacteria isolated from larval oral secretions to wounded plants, Chung and coauthors [22] demonstrated that microbial symbionts belonging to the genera *Stenotrophomonas*, *Pseudomonas*, and *Enterobacter* were responsible for host plant defense suppression in Colorado potato beetles (*Leptinotarsa decemlineata*). We identified several representatives of all three genera in *E*. *varivestis* regurgitant: 6 *Stenotrophomonas*, 55 *Pseudomonas*, and 12 *Enterobacter*. Jasmonic acid defense-suppressing Enterobacteriaceae-1 (*Serratia*) identified in fall armyworm oral secretions [23], were also found in *E*. *varivestis* regurgitant. Overall, the four phyla most represented in our dataset (Firmicutes, Actinobacteris, Bacterioidetes, and Proteobacteria) are also the most commonly associated with insect species [80]. Many of the other microbes identified are commonly associated with the soybean phyllosphere (e.g., Actinobacteria, Bacteroidetes, and Proteobacteria) [81]. Future studies are needed to explore the impact of these microbes on soybean-*E*. *varivestis* interactions, and to better disentangle the relative contributions of the regurgitant bacterial communities on overcoming plant defense strategies.

### Role of regurgitant of beetle-borne viruses specificity

Beetle-bornes viruses can only be transmitted plant to plant by beetles, and it is thought that the beetle’s regurgitant plays a significant role in this specificity. Previous research demonstrated that non-beetle-transmissible plant viruses lost their infectivity when mixed with the regurgitant of leaf-feeding beetles, whereas beetle-transmissible remained infectious [11,27,28]. To investigate this further, we assayed virus-vector specificity of *E*. *varivestis* and two prevalent soybean viruses: an aphid vectored virus not known to be transmissible by beetles, *Soybean mosaic virus* (SMV), and a virus naturally vectored by the beetle, *Bean pod mottle virus* (BPMV). Moreover, two different leaf wounding approaches were implemented: mechanical inoculation via an abrasive, as well as gross wounding technique that more naturally simulated wounding induced by beetle feeding. Table 1 shows the effect of beetle regurgitant on virus transmission for the different wounding techniques and virus:regurgitant combinations deposited at the leaf wounding sites.

**Table 1:** Impact of regurgitant on transmission of a beetle-borne virus (*Bean pod mottle virus*, BPMV) and a non-beetle transmissible virus (*Soybean mosaic virus*, SMV) using two different leaf inoculation techniques (mechanical, M and gross wounding, G). For each inoculum/virus combination, 30 experimental plants were assayed. The percentage of virus-infected plants is shown and the number of infected plants is indicated in parenthesis. Statistical significance between treatments was tested using a Marascuilo procedure (see methods), different superscript letters denote statistically different treatments (*P* < 0.05).

Virus	Leaf Wounding	Ratio of beetle regurgitant to purified virus in inoculum mixture					
		1:1	1:5	1:10	1:15	1:20	1:500
**BPMV**	G	93.3% ^a^ (28)	90% ^a^ (27)	90% ^a^ (27)	86.7% ^a^ (26)	93.3% ^a^ (28)	90% ^a^ (29)
	M	90% ^a^ (27)	96.7% ^a^ (29)	100% ^a^ (30)	90% ^a^ (27)	93.3% ^a^ (28)	90% ^a^ (29)
**SMV**	G	0% ^b^ (0)	3.33% ^b^ (1)	0% ^b^ (0)	6.67% ^b^ (2)	0% ^b^ (0)	86.7% ^a^ (28)
	M	0% ^b^ (0)	0% ^b^ (0)	0% ^b^ (0)	6.67% ^b^ (2)	0% ^b^ (0)	90% ^a^ (29)

Our results indicated that *E*. *varivestis* regurgitant effectively suppresses infection of the non-beetle-borne SMV when the inoculum is diluted ≤1:20 (*P* < 0.05 across all dilutions and wounding approaches). At 1:500 dilution, the inhibitory effects of the regurgitant were negligible. On the other hand, the regurgitant did not impact the infectivity of the beetle transmissible BPMV, regardless of concentration. The results were consistent across virus/inocula combinations irrespective of leaf wounding technique implemented. Overall, <2% of plants inoculated with a mixture of SMV and *E*. *varivestis* regurgitant diluted ≤20-fold became infected, whereas 94% of plants of the BPMV-inoculated plants became infected.

Our findings show some disagreement with that of [11]. Indeed, the authors found only gross wounding selectively inhibited infection of the non-beetle-borne *Tobacco ringspot virus* (TRSV). Inoculation by mechanical inoculation completely suppressed infection by both TRSV and the beetle-borne *Southern bean mosaic virus* at dilutions less than 1:320. The discrepancies could be attributed to deviations among studies in leaf-rub inoculation techniques, buffer composition, or could allude to some variability in specificity among Coleopteran viruses.

It appears very likely that the beetle regurgitant contains factor(s) that selectively prevent infection of plants by non-beetle-transmissible viruses. Previous work has shown the inability of virus particles to infect hosts is not due to inactivation since virus particles regained infectivity when purified away from the regurgitant [26]. This suggests that the inhibitor(s) in the regurgitant directly impact the host or alters the interactions between the virus and host in some capacity. Gergerich and coauthors [59] provided evidence that the high RNase activity in beetle regurgitant plays a role in the selective inhibition. This is substantiated by a study on *E*. *varivestis*, indicating that ribonucleases in the beetle’s regurgitant may boost plant defenses and ultimately virus infection [82]. Still, we have shown beetle regurgitant is a highly complex molecular and biological substance that contains thousands of factors that could potentially influence vector-virus specificity. Moreover, some of these factors could even contribute to differences in vector competence found among beetle species and among individuals of the same species [83]. Studies have been far more numerous characterizing the salivary transcriptomes/proteomes of sap-sucking insects, and point to an exhaustive list of potential effectors [12–21]. Future studies are needed to disentangle the functional roles of the regurgitant components and how they relate to virus transmission.

### Conclusions

This study presents the first comprehensive high-throughput regurgitome of a beetle species. Leaf-feeding beetles, such as *E*. *varivestis*, deposit regurgitant onto wounded leaves during feeding. Analogous to the saliva of sap-sucking insects, it has been speculated that these oral secretions perform vital roles in the feeding process by initiating digestion and suppressing anti-herbivory host defenses. Moreover, the regurgitant is also thought to play a unique role in the remarkable specificity of beetle-transmissible viruses. Our study demonstrates that the regurgitant of *E*. *varivestis* is surprisingly complex, comprised of an impressive arsenal of putative extracellular proteins and microbes. Further, we show that the regurgitant is fundamental to the specificity of beetle-transmissible viruses. Ultimately, this study provides an exhaustive list of candidates, some of which could play important roles in plant-insect interactions and virus transmission.

## Supporting information

S1 Table SignalP information for the 1,555 *Epilachna varivestis* peptide sequences predicted to have a secretion signal. (XLSX)

S2 Table Functional annotation of the 992 *Epilachna varivestis* contigs predicted to have a secretion signal. (XLSX)

S3 Table The 1,230 bacterial species representing 577 genera found in *Epilachna varivestis* regurgitant. (XLSX)
